# Genome-Wide Identification of *Hsp90* Gene Family in Perennial Ryegrass and Expression Analysis under Various Abiotic Stresses

**DOI:** 10.3390/plants10112509

**Published:** 2021-11-19

**Authors:** Charlotte Appiah, Zhong-Fu Yang, Jie He, Yang Wang, Jie Zhou, Wen-Zhi Xu, Gang Nie, Yong-Qun Zhu

**Affiliations:** 1 Department of Forage Science, College of Grassland Science and Technology, Sichuan Agricultural University, Chengdu 611130, China; charlotte27appiah@gmail.com (C.A.); yangzf211@163.com (Z.-F.Y.); he13440090515@163.com (J.H.); wangyll5123@163.com (Y.W.); zhoujie1@stu.sicau.edu.cn (J.Z.); 2 lnstitute of Agricultural Resources and Environment, Sichuan Academy of Agricultural Sciences, Chengdu 610066, China; xuwenzhi_herb@126.com

**Keywords:** perennial ryegrass, *Hsp90*, abiotic stress, expression profiles, phylogenetic analysis

## Abstract

The heat shock protein 90 (*Hsp90*) is a protein produced in plants in response to stress. This study identified and analyzed *Hsp90* gene family members in the perennial ryegrass genome. From the results, eight *Hsp90* proteins were obtained and their MW, pI and number of amino acid bases varied. The amino acid bases ranged from 526 to 862. The CDS also ranged from 20 (*LpHsp0-4*) to 1 *(LpHsp90-5*). The least number of CDS regions was 1 (*LpHsp90-5*) with 528 kb amino acids, while the highest was 20 (*LpHsp90-4*) with 862 kb amino acids, which showed diversity among the protein sequences. The phylogenetic tree revealed that *Hsp90* genes in *Lolium perenne*, *Arabidopsis thaliana*, *Oryza sativa* and *Brachypodium distachyon* could be divided into two groups with five paralogous gene pairs and three orthologous gene pairs. The expression analysis after perennial ryegrass was subjected to heat, salt, chromium (Cr), cadmium (Cd), polyethylene glycol (PEG) and abscisic acid (ABA) revealed that *LpHsp90* genes were generally highly expressed under heat stress, but only two *LpHsp90* proteins were expressed under Cr stresses. Additionally, the expression of the *LpHsp90* proteins differed at each time point in all treatments. This study provides the basis for an understanding of the functions of *LpHsp90* proteins in abiotic stress studies and in plant breeding.

## 1. Introduction

Improving stress tolerance is among the major efforts of breeding advancement in cool season grass species [1]. Perennial ryegrass (*Lolium perenne*) is one of the major species of forage and turf grasses extensively planted in warm temperate to subtropical regions around the world, because it is to plant, has better tolerance to abiotic stresses, and requires low maintenance [2]. However, achieving the potential yield after cultivation of perennial ryegrass is limited due to exposure to abiotic stresses in cultivated lands [3]. Abiotic stresses such as cold, drought, salinity, freezing, high light intensity and heat cause cell injury resulting in secondary stresses such as osmotic and oxidative stresses that critically impact the quality and yield of the perennial ryegrass plant [4,5,6]. Moreover, the impact of stresses in *Lolium perenne* is evident in limiting cultivation, leaf appearance, seed emergence, reducing dry matter by up to 25% and causing plant death [7,8,9,10]. The response of plants to heat shock is similar to that of other organisms when exposed to adverse stress conditions, producing highly conserved stress proteins called heat shock proteins (*Hsp*) [11,12,13].

Heat shock proteins are expressed in response to stresses and are highly conserved at both the cellular and organismic levels [14,15]. Generally, plants have five major classifications of *Hsp*s based on their molecular size: *Hsp100*, *Hsp90*, *Hsp70*, *Hsp60* and small *Hsp* (*smHsp*) [16]. One of the most numerous proteins in the cytoplasm of prokaryotic and eukaryotes belongs to the *Hsp90* family, constituting 1–2% of the total protein level [17]. *Hsp90* is an important constituent of cells at developmental stages under normal conditions, aiding in protein translocation, folding and degradation [18]. Furthermore, the heat shock protein transcript is significantly upregulated under heat stress [19], and also some stresses are known to induce synthesis and expression of heat shock proteins in cells [20]. Under stress conditions, *Hsp90* functions as a homeostatic agent by reestablishing the damaged normal protein structure [5,21]. *Hsp90* has important functions in both animals and plants. *Hsp90* is abundantly expressed in the cytoplasm in soluble form at normal temperatures in yeast, fruit flies, and vertebrate families, while it accumulates rapidly in the nucleus under heat shock conditions [22]. *Hsp90* has been well characterized in *Oryza sativa*, *Arabidopsis thaliana* and *Solanum lycopersicum* [18,19,20]. *Hsp90* was also recently identified in *Aeluropus littoralis*, *Hordeum vulgare*, *Camellia sinensis* and *Cucumis sativus* [23,24,25,26].

*Hsp90* is an ATP-regulated dimeric chaperone mainly consisting of three highly conserved domains: C-terminal domain of about 25 kDa that binds to the substrate, the 35 kDa intermediate domain, and the 12 kDa N-terminal domain of the ATP-binding site. *Hsp90* cooperates with other chaperones to form a multiprotein chaperone complex in order to play its role [27]. *Hsp90* can be further divided into five subgroups in accordance to the source and subcellular localization. The subgroups are namely *Hsp90A*, *Hsp90B*, *Hsp90C*, TRAP (TNF receptor-associated protein) and HTPG (high temperature protein G) [28]. *Hsp90A* contains no signal peptide and is located in the cytoplasm. The main subtypes include *Hsp90α* (inducible form) and *Hsp90β* (constitutive form), which are the result of gene duplications about 500 million years ago [25,26]. *Hsp90B*, *Hsp90C* and TRAP are located in the endoplasmic reticulum, chloroplast and mitochondria (Animalia), respectively, because they contain signal peptides. HTPG refers to the *Hsp90* of prokaryotes and is distributed in most bacteria [24,28]. Several biological variants of the *HSP90* gene have also been well characterized in some plants. In *Arabidopsis*, *AtHsp90-5* is important for chloroplast biogenesis and embryogenesis [29,30]. In *Brassica napus*, *Hsp90* plays a vital role in the processes of seed development and germination, while in cotton *Hsp90* has been found to play a crucial role in cotton fiber differentiation and development by maintaining cellular homeostasis [31,32,33,34,35,36]. *Hsp90*s, as housekeeping proteins in plants, can be induced by various abiotic and biotic stresses [37]. Expression of *Hsp90* in *Arabidopsis thaliana* is developmentally regulated and is responsive to abiotic stresses, phytohormones, and light and dark transitions [19,21,38,39,40]. The overexpression of *AtHsp90-2*, *AtHsp90-5*, and *AtHsp90-7* reduces tolerance to salt and drought stresses whiles improving tolerance to high concentrations of Ca^2+^ [39]. Moreover, the overexpression of *Hsp90-2* in *Arabidopsis thaliana* may inhibit the transcription of HsfA2, and HsfA2 expressed under the inhibition of *Hsp90-2* contributes to the resistance to oxidative stress [41]. Similarly, an *Hsp90* inhibitor produced by root-peripheral fungi may inhibit plant growth and development, but also increase the resistance of *Arabidopsis* to high temperatures [42]. The *Hsp90* complex in *Arabidopsis* directly regulates the activity of resistance proteins and plays a key role in disease resistance as well [43]. The induction of ABA responsive genes is delayed by overexpression of cytosolic *AtHsp90-2*, but is hardly affected by overexpression of *AtHsp90-5* and *AtHsp90-7* under conditions of salt and drought stress, which implies that different cellular compartment localized *Hsp90*s in *Arabidopsis thaliana* might contribute to responses to abiotic stresses by different functional mechanisms, probably through ABA- or Ca^2+^ dependent pathways [39]. *OsHsp90-2* and *OsHsp90-4* were also found to be up-regulated to drought, cold, heat and salt stresses [38]. The resistance of tobacco leaves to mosaic virus increases because of the interaction of *Hsp90* with RAR1 and TIR-NB-LRR in tobacco leaves [44]. In tobacco, *NtHsp90s* were strongly induced by heat stress, while weakly activated by ABA treatment, with expression pattern analysis indicating that *NtHsp90-4*, *NtHsp90-5*, and *NtHsp90-9* were induced by various abiotic stresses. The expression level of *UpHsp90* in *Ulva pertusa* is notably positively regulated by the change in temperature difference between day and night, but it was almost unaffected under long-term treatment with heavy metal stress [45]. In potatoes, *Hsp90*s may be related to the color of potato tuber chip [46]. *Hsp90*s play vital roles in the growth of tumor cells. For example, geldanamycin can specifically interact with the ATPase active site of *Hsp90*, preventing the binding of *Hsp90* and ATP, and finally achieve the purpose of inhibiting tumor [47]. Using interference technology, the expression level of *Hsp90* was reduced, and it was found that the division rate of U937 cells was significantly reduced [48].

According to previous studies, eight *Hsp90* genes were identified in *Oryza sativa*, seven in *Arabidopsis thaliana*, eight in *Brachypodium distachyon*, ten in *P. trichocarpa*, 21 in *Nicotiana tabacum*, seven in *Solanum lycopersicum*, and twelve in *Zea mays* [40,49,50,51]. However, the identification of the perennial ryegrass *Hsp90* gene family has not been studied yet. The completion of genome-wide sequencing of perennial ryegrass will provide the necessary information for data mining of *Hsp90* at the whole genome level. In this study, we performed a genome-wide survey of *Hsp90* in the perennial ryegrass genome database, and a complete overview was reported on gene structure and phylogenetic and conserved motif characteristics. Additionally, the expression levels of the *LpHsp90* genes under various abiotic stresses were studied. The results will be helpful for further study of the functional characteristics of *Hsp90* genes in response to abiotic stress in perennial ryegrass.

## 2. Results

### 2.1. Identification of LpHsp90 Genes in Perennial Ryegrass

Eight *LpHsp90* genes were identified after the removal of redundant sequences from the genome database of perennial ryegrass. LpHps90 proteins were renamed according to their chromosomal locations; that is, *LpHsp90-1* to *LpHsp90-8*. *LpHsp90* sequences obtained varied in length, which ranged from 528 (*LpHsp90-5*) to 862 (*LpHsp90-4*) amino acids, with an average of 779. The pI values ranged from 4.89 (*LpHsp90-5*) to 5.57 (*LpHsp90-4*) and with a MW ranging from 61214.61 kd (*LpHsp90-5*) to 96712.15 kd (*LpHsp90-4*). The *LpHsp90s* were highly cytoplasmic (*LpHsp90-1, 2, 3, 5, 7 and 8*) with the exception of *LpHsp90-4* and *LpHsp90-6*, which were nuclear and ER subcellular localized, respectively (Table 1). Moreover, the analysis of the cis-acting elements of perennial ryegrass *Hsp90* genes showed that plant hormone responsiveness was identified, implying that *LpHsp90* genes might be involved in various plant stress-responsive pathways and closely related to the function of plant hormones such as abscisic acid, gibberellin and methyl-jasmonate (Table S1).

### 2.2. Phylogenetic Analysis and Multiple Sequence Alignment

**Figure 1:**
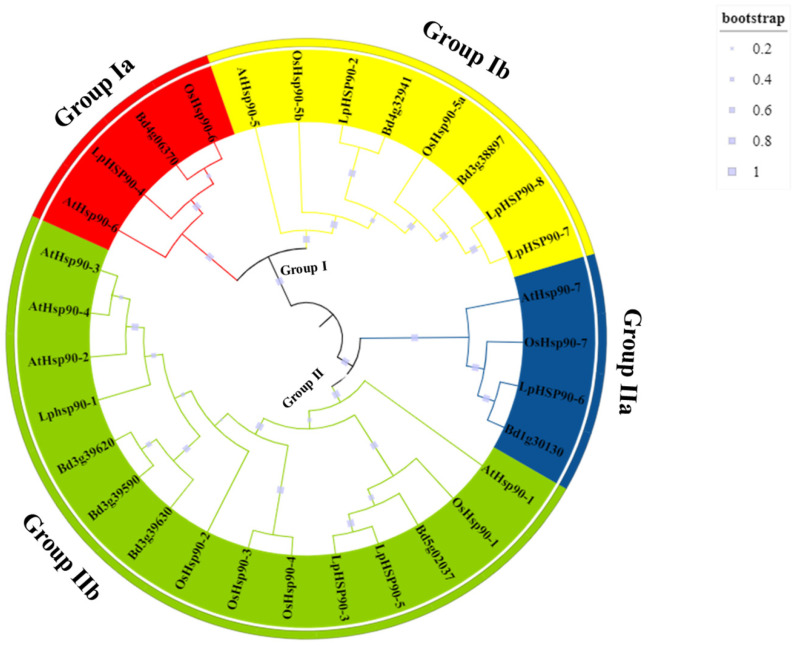
Unrooted phylogenetic tree representing relationships among the *Hsp90* protein sequences of *Lolium perenne*, *Arabidopsis thaliana*, *Brachypodium distachyon* and *Oryza sativa*. The tree was divided into two main groups (I and II) and further divided into subgroups (Ia, Ib, Iia and Iib). Ia had 4 members, and Ib had 8 members; Iia had 4 members and Iib had 15 members.

The *Hsp90* protein sequence alignments of *Lolium perenne*, *Oryza sativa*, *Arabidopsis thaliana* and *Brachypodium distachyon* were used to construct a phylogenetic tree employing the maximum-likelihood method with 1000 bootstraps to explore the evolutionary relationship among the plant species using MEGA6 (Figure 1). The *Hsp90* protein sequences were classified into two main groups (group I and II), and each group was further divided into two subgroups (Ia, Ib, IIa and Iib). The group Iib (15 members) had the largest number of members, followed by group Ib (8 members). It was also seen that groups Ia and Iia had 4 members each. Additionally, the phylogenetic tree showed that there was high similarity among cytosolic *Hsp90s* and less similarity among the organelle-localized members.

The phylogenetic tree analysis showed that there were five pairs of paralogs within species, of which two were from *Lolium perenne* (*LpHsp90-7* and *LpHsp90-8*, *LpHsp90-3* and *LpHsp90-5*), one pair in *Oryza sativa* (*OsHsp90-3* and *OsHsp90-4*), one pair in *Brachypodium distachyon* (*Bd3g39620* and *Bd3g39590*) and one pair in *Arabidopsis thaliana* (*AtHsp90-2* and *AtHsp90-3*) (Figure S1) [50]. There were three orthologous gene pairs among the species (*Bd4g06370* and *LpHsp90-4*, *Bd1g30130* and *LpHsp90-6*, *Bd4g32941* and *LpHsp90-2*) [51]. The orthologous and paralog genes may predict the functions and characteristics of the *LpHsp90* genes in the evolutionary relation with *Arabidopsis thaliana*, *Brachypodium distachyon* and *Oryza sativa* [52].

### 2.3. Conserved Motif and Gene Structure Analysis of LpHsp90 Proteins

**Figure 2:**
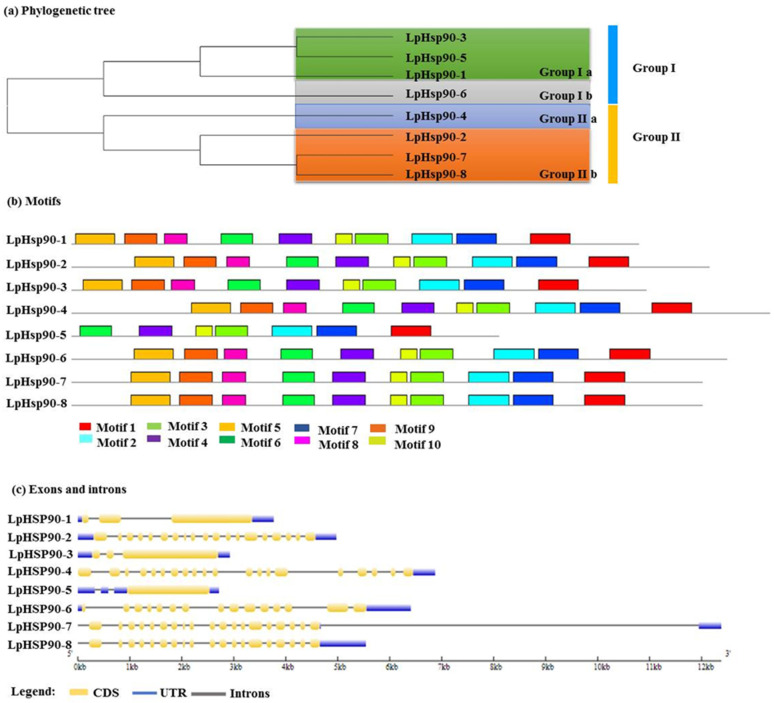
Phylogenetic relationship, gene structure and motif composition of *Hsp90* genes in perennial ryegrass. (**a**) A multiple sequence alignment of the full length of *LpHsp90* protein sequences was executed using Clustal W, and a maximum-likelihood phylogenetic tree with 1000 bootstraps was constructed using MEGA 6.0 software. The four subgroups were marked with different colors. (**b**) Schematic representation of the conserved motifs obtained using MEME online tool in *LpHsp90* proteins. Different motifs are represented by colored boxes of corresponding colors. The grey lines represent the non-conserved. (**c**) The exon/intron structure of the *LpHsp90* protein sequences was obtained employing the online tool GSDS.

A maximum likelihood phylogenetic tree was constructed using eight protein sequences of *LpHsp90* (Figure 2a). The *LpHsp90* sequences were divided into two main subgroups according to the bootstrap values and motif compositions. Ten conserved motifs were identified using the online software MEME (Figure 2b). It was observed that all *LpHsp90* protein sequences in the same group had similar motif compositions and positionings. All motifs were arranged in the order of motif -5, motif-9, motif-8, motif-6, motif-4, motif-10, motif-3, motif-2, motif-7 and motif-1 apart from *LpHsp90-5*. In group Ia, it was observed that *LpHsp90-5* had only seven motifs with the exclusion of motif-5, motif-9 and motif-8, but was similar in motif position with *LpHsp90-1* and *LpHsp90-3*, which may be due to evolutionary change. Additionally, *LpHsp9-2*, *LpHsp90-7* and *LpHsp90-8* in group Iib were similar in motif composition and positioning. Details of motif logo and consensus are listed in supplement file Figure S2. Besides, three heat shock genes *(LpHsp90-1*, *LpHsp90-3* and *LpHsp90-5*) containing the C-terminal EEVD motif were predicted that they functionally interacted with other family members and were seen to be highly similar (Figure S3).

The analysis of the exon-intron structure of *Hsp90* protein sequences of perennial ryegrass may provide insights into the evolution of the *LpHsp90* gene family [53]. The online software GSDS tool was used to obtain the exon-intron structure of the *Hsp90* protein sequences. Figure 2c revealed a coding sequence of *LpHsp90* protein interrupted by introns. The least number of CDS regions was one (*LpHsp90-5*), with 528 kb amino acids, while the highest was twenty (*LpHsp90-4*), with 862 kb amino acids, which showed diversity among the protein sequences. *LpHsp90-7* had the longest gene structure due to the length of the intron, although *LpHsp90* had the highest number of CDS regions. Moreover, comparing *LpHsp90-8* and *LpHsp90-7*, they were different in gene structure but had the same MW, pI and number of amino acids.

### 2.4. Expression Profile of LpHsp90 in Response to Abiotic Stresses

**Figure 3:**
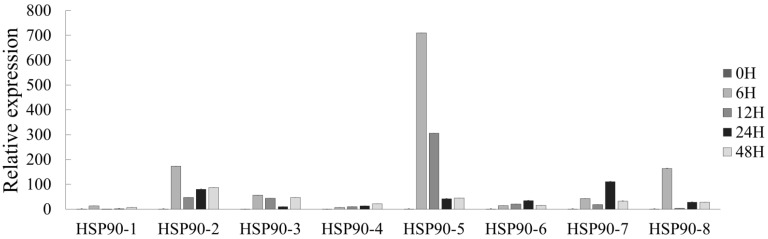
Expression patterns of *LpHsp90s* in response to heat. The values are the mean values of expression (*n* = 3).

**Figure 4:**
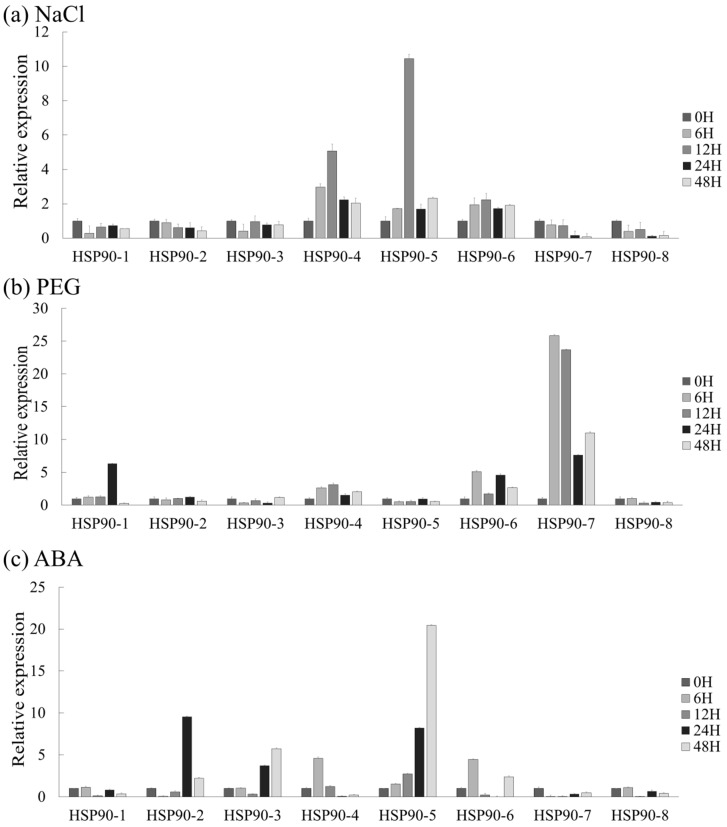
Expression patterns of *LpHsp90s* in response to (**a**) NaCl (**b**) PEG and (**c**) ABA treatment. The values are the mean values of expression (*n* = 3).

**Figure 5:**
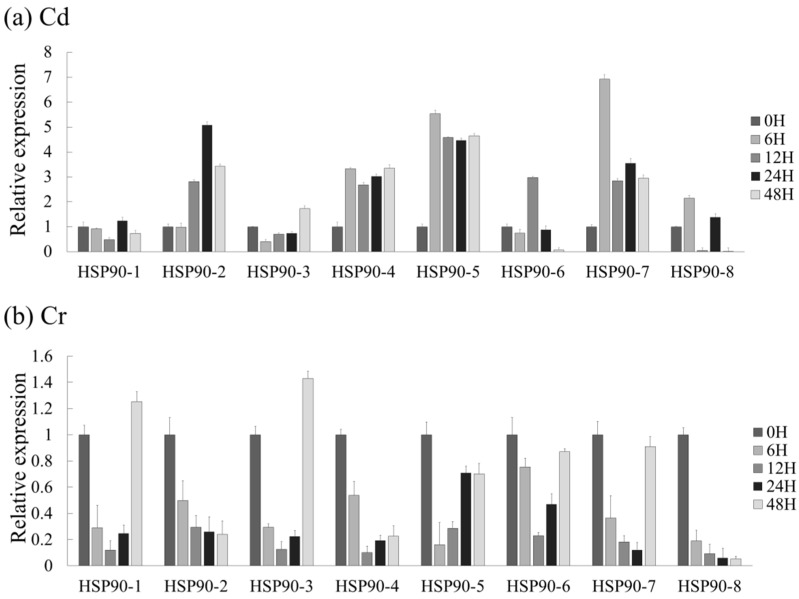
Expression patterns of *LpHsp90s* in response to (**a**) Cd and (**b**) Cr treatment. The values are the mean values of expression (*n* = 3).

Most plants have mechanisms for defense against stress, and *Hsp90* genes are known to be expressed in response to these abiotic stresses [31]. To analyze the expression pattens of *LpHsp90* under abiotic stress, eight *LpHsp90* proteins were analyzed using the qRT-PCR technique. As shown in Figure 3, Figure 4 and Figure 5, different expression patterns were observed under ABA, cadmium (Cd), chromium (Cr), salt (NaCl), heat and PEG induced abiotic stresses. It was observed that *LpHsp90* gene regulation was consistent across all stresses at the 0-h time point.

*LpHsp90* was highly expressed under heat stress as compared to other treatments. *LpHsp90-5* had the highest expression value under heat stress, which was recorded at two time points, 6 h and 12 h (Figure 3). At 6 h, it was also observed that *LpHsp90-2* and *LpHsp90-4* were substantially expressed.

Under salt stress, *LpHsp90-5* was significantly expressed at 12 h (Figure 4a). *Lphsp90-4* was seen to be expressed at 12 h also. Considering drought induced by PEG treatment at 6 h, *LpHsp90-7* was highly induced at 12 h (Figure 4b). *LpHsp90-5* and *LpHsp90-8* were not expressed at any time point. It was observed that the expression patterns of *LpHsp90* proteins were irregular at the various time points, owing to the fact that an increase in expression at 6 h may decrease at 12 h or an increase in expression at 24 h may decrease at 48 h. ABA treatment up-regulated *LpHsp90-5* consistently across all time points, while *LpHsp90-7* was not induced by ABA across all time points (Figure 4c). Some *LpHsp90* genes (*LpHsp90-1*, *LpHsp90-2*, *LpHsp90-3*, *LpHsp90-4*, *LpHsp90-6* and *LpHsp90-8*) were seen to have an undulating expression pattern, thus their expression may be affected by the longevity of exposure to ABA stress.

Under Cr treatment, *LpHsp90-7* was significantly expressed at 6 h but was seen to have reduced with longer time of exposure. *LpHsp90-5* was also observed to be expressed at the 6 h time point. Further, *LpHsp90-2* was highly expressed at 24 h but reduced at 48 h, while *LpHsp90-3* was induced at 48 h under Cd stress (Figure 5a). Cr stress induced the weakest expression, although *LpHsp90-1* and *LpHsp90-3* were induced at the 48 h time point while all of the remaining six *LpHsp90* genes were fairly activated compared to other treatments.

Generally, *LpHsp90-1*, *LpHsp90-3*, *LpHsp90-4* and *LpHsp90-6* were induced under five stresses, while *LpHsp90-7* and *LpHsp90-8* were induced under only two stresses. *LpHsp90* showed the highest expression at 6 h (*LpHSP90-5*, under heat stress), and the lowest expression was recorded at 48 h (*LpHsp90-8*, under Cd stress). A heatmap showing the expression of each *LpHsp90* gene was drawn under all six abiotic treatments (Figure S4).

## 3. Discussion

The heat stress proteins have been classified based on their molecular weights into *Hsp100*, *Hsp90*, *Hsp70*, *Hsp60* and *smHsp* [54]; among them, *Hsp90* was known to be important and highly conserved [55,56]. *HSP*s were first identified in the salivary gland chromosomes of Drosophila larva [57]. Later, other studies found organisms that produced a series of proteins of different sizes, known as *HSP*s, in response to increased temperature [58]. The study of *Hsp90* genes proved that they were not only related to stress signal transduction in plants, folding of receptors, transcription factors and kinases and physiological processes [51,52,53,58], but also to assisting cell survival under stresses [59]. Studies have found out that besides high temperatures, abiotic stresses such as drought, salinity, heavy metals and ABA could induce the production of *Hsp90* in plants [60,61]. *Hsp90* proteins have been identified in plants and differences in their numbers are attributable to their genome sizes. Seven *Hsp90* proteins were identified in *Arabidopsis thaliana* [38], 10 in *P. trichocarpa* [62], 21 in *Nicotiana tabacum* [63], seven in *Solanum lycopersicum* [64], seven in *Oryza sativa* [65], eight in *Brachypodium distachyon* and 12 in *Zea mays* (Maize genome database, http://www.maizegdb.org, accessed on 1 August 2020). As perennial ryegrass is one of the major species of forage and turf grasses extensively planted in warm temperate to subtropical regions around the world, it was expedient to obtain genes that would further aid in its improvement. Therefore, it was necessary to identify *Hsp90* genes related to various abiotic stresses in perennial ryegrass. The identification of Lolium perenne *Hsp90* will provide more insights and fundamental information into genetic improvements in response to stressful conditions in other plants.

In this study, eight *Hsp90* protein sequences were identified in the perennial ryegrass genome database. The *Hsp90*s may play a role in the physiological and environmental stability of perennial ryegrass. The *LpHsp90*s identified had different biophysical and chemical properties, which indicated diversity among the *LpHsp90* protein sequences. The isoelectric point of the *LpHsp90* protein sequences ranged from 4.89 to 5.57, making them acidic, which is consistent with *Hsp*s found in *Arabidopsis thaliana*, *Solanum lycopersicum* and other plants [66]. Phylogenetic analysis between *Hsp90s* of *Lolium perenne*, *Arabidopsis thaliana*, *Oryza sativa* and *Brachypodium distachyon* divided the *Hsp*s into two groups, consistent with other studies performed [67]. Additionally, phylogenetic analysis aided in the identification of five paralog gene pairs among the plant species. This may imply that most species expanded according to their own species-specific approach during evolution of the *Hsp90* family [50,68]. This finding is consistent with gene families found in cereals such as rice and also in *Nicotiana tabacum* [50,69]. The structure of proteins is known to determine the function they may perform [50,67]. According to the study, *LpHsp90* protein sequences had different gene structure. The number of introns is mostly related to the sensitivity of gene transcription regulation, thus the lesser the number of introns, the more likely that a plant has the ability to adapt to different developmental and environmental stimuli [70]. Theoretical pI and number of amino acids between *LpHsp90-7* and *LpHsp90-8* do not imply they may perform the same function due to the difference in their gene structure. Furthermore, the predicted cis-acting elements from Plantcare stated the likelihood of *LpHsp90-7* to be involved in responsiveness to MeJA and gibberellin.

The study of the expression of *Hsp90* proteins in response to abiotic stresses has been undertaken in various plant species. In this study, it was seen that most *LpHsp90* protein genes were induced under most of the stresses. Heat stress recorded the highest expression in all *LpHsp90* proteins, which is consistent with studies in populous and *CsHSP90* genes, although the expression levels of certain genes were brief and slightly decreased at individual time points [25,62]. The comparatively lowest level of expression was recorded under Cr stress. *LpHsp90-5* was observed to be highly expressed under ABA, heat and salt stress, but these were at different time points. In ABA, there was a rapid rise in the expression of *LpHsp90-5*, with the highest expression level recorded at 48 h. Unlike ABA, NaCl stress induced high expression in *LpHsp90-5* at 12 h but with a rapid reduction at 24 h and 48 h. Under heat stress, there was also a decrease in expression after peaking at 6 h. Additionally, *LpHsp90-7* was highly expressed under PEG and Cd stress, and these were also recorded at different time points. *LpHsp90-1* and *LpHs90-3* were the only *Hsp90* proteins expressed under Cr heavy metal stress. This could imply that *LpHsp90-5* could be an *LpHs90* gene of interest in ABA, heat, and salt stress; *LpHsp90-7* to PEG and Cd; and *LpHsp90-3* and *LpHsp90-1* to Cr. Analysis of *AtHsp90-5* and *AtHsp90-6* expression revealed that the former is mildly induced by heat shock and that the latter is barely induced by heat shock; this was also observed in *LpHsp90-5* in this study. Comparatively, *LpHsp90-2* expression levels increased modestly with heat stress; this was also observed in *AtHsp90-2*, the levels of which were mildly increased after treatment with NaCl or heavy metals [39]. Therefore, further studies may be carried out to explain the functions of *LpHsp90-7*, *LpHsp90-5*, *LpHsp90-3* and *LpHsp90-1* in relation to these stresses. Moreover, the various time points of the expression of the LpHsp90 proteins should also be further investigated, since the effects of stress are influenced by intensity and longevity. It was also observed that the paralogous pairs (*Lphsp90-5* and *LpHsp90-3*, *LpHsp90-7* and *LpHsp90-8*) had different expression profiles under the various abiotic stresses.

These results showed that *LpHsp90s* were induced under ABA, PEG, Cr, Cd NaCl and heat stress, and individual *LpHsp90s* may have different regulatory patterns that reflect their potential roles in the response to different abiotic stresses. Heat stress induced the highest response of all stresses, indicating that the *LpHsp90* protein was very sensitive to heat stress.

## 4. Materials and Methods

### 4.1. Identification of LpHsp90 Genes in Perennial Ryegrass

*Arabidopsis thalianaHsp90* protein sequences were downloaded from the TAIR databases (http://www.arabidopsis.org/, accessed on 1 April 2020) [39]. The protein sequences of *Arabidopsis thaliana* *Hsp90* genes were used as queries to perform BLASTP against the genome resource of perennial ryegrass, which was downloaded from the Perennial Ryegrass Genome Sequencing Project (http://185.45.23.197:5080/ryegrassgenome, accessed on 1 April 2020) [71], followed by the removal of redundant proteins. Subcellular localization of *LpHsp90* proteins was predicted by using CELLO (http://cello.life.nctu.edu.tw/, accessed on 1 August 2020). The physical and chemical parameters of the *LpHsp90* protein sequences were obtained from ProtParam (https://web.expasy.org/protparam, accessed on 1 August 2020).

### 4.2. Phylogenetic Analysis and Multiple Sequence Alignment

A multiple sequence alignment of *Lolium perenne*, *Arabidopsis thaliana*, *Brachypodium distachyon* and *Oryza sativa* *Hsp90* protein sequences was analyzed using ClustalW [72]. *Oryza sativa Hsp90s* were obtained from the Rice Genome Annotation Project (http://rice.plantbiology.msu.edu/, accessed on 1 April 2020). Brachypodium distachyon were obtained from the Phytozome link in Brachypodium distachyon Assembly and Gene Annotation in ensemble (http://www.phytozome.net/, accessed on 1 April 2020). A maximum-likelihood (ML) phylogenetic tree was constructed with MEGA version 6.0 employing 1000 bootstraps in order to examine the evolutionary relationships among the *Hsp90* family of *Lolium perenne*, *Arabidopsis thaliana*, *Brachypodium distachyon* and *Oryza sativa* [73,74].

### 4.3. Exon-Intron Structure, Conserved Motif, Characteristics Analysis of LpHsp90

The exon-intron structure of *Hsp90* genes was obtained using the online Gene Structure Display Server v2.0 (GSDS: http://gsds.cbi.pku.edu.cn, accessed on 1 October 2020) [75] with the giff3 file. The MEME program (Multiple Expectation Maximization for Motif Elicitation (http://memesuite.org/tools/meme/, accessed on 1 October 2020) [76] was used to identify the conserved motifs in the *LpHsp90* protein sequences.

### 4.4. Plant Materials, Growth Conditions and Stress Application

Viable seeds of *Lolium perenne* cv ‘Mathilde’ were grown in a container with dimensions 20 cm × 15 cm × 10 cm. The container was filled with quartz sand and distilled water to one-third of the volume, after which the seeds were evenly spread on the sand to avoid clustering of plants. The container with the seeds was moved to the growth chamber. Plants were grown at a temperature of 20 °C and 15 °C (12 h day/12 h night) at 70% relative humidity and 750 µmol·m^−2^·s^−1^ PAR illumination. After 14 days of germination, Hoagland solution dissolved in distilled water following the manufacturer’s protocol was used to supply plants with nutrients for 46 days. The plants were separated into groups of five for the various stress applications.

For salt stress, sodium chloride (NaCl) was used at a concentration of 250 mM by dissolving NaCl in Hoagland solution. For heavy metal stresses, chromium (Cr) (K2Cr2O7) and cadmium (Cd) (CdCl_3_·6H_2_O) were used at a concentration of 300 mg/L and 200 mg/L, respectively. Twenty-percent polyethylene glycol 6000 (PEG) was used to stimulate drought stress after dissolving in Hoagland solution. For heat stress, plants were transferred to a growth chamber and subjected to a temperature of 38 °C/30 °C (day/night) with 12 h light/ 12 h dark photoperiod and all other conditions remaining constant, as per the early growth stage. Plants were subjected to 100 mM ABA for the exogenous abscisic acid (ABA) application. Collection of plant leaf samples was performed in time frames of 0 h, 6 h, 12 h, 24 h, 48 h and 72 h after each stress application with three biological replications. Plant leaf samples were collected into a 1.5 mL tube using a pair of surgical scissors, which were intermittently cleaned with ethanol after every stress sample collection to avoid contamination. Approximately 1 g of collected leaves was quickly frozen using liquid nitrogen and stored in a freezer at −80 °C.

### 4.5. RNA Isolation, cDNA Synthesis and Quantitative Real-Time PCR Expression Analysis

For all RNAs isolated from leaf samples, HiPure Plant RNA Mini Kit (Magen Biotech Co. Ltd., Guangzhou, China) was used following the manufacturer’s protocol. A Nanodrop ND-2000 spectrophotometer (Nano-Drop Technologies, Wilmington, DE, USA) was used to determine RNA concentration, purity, and integrity, followed by 1% agarose gel electrophoresis. M5 Super plus qPCR RT kit with gDNA remover (Mei5 Biotechnology, Co., Ltd. Beijing, China) were used for the RNA reverse transcript. The qRT-PCR technique was used to validate the expression of *LpHSP90* genes in the various stress treatments and the specific primers obtained as designed by Premier 3.0 (Table 2). A 10 µL mixture was prepared for each sample, containing 5 μL of abm^®^ EvaGreen 2X qPCR Master Mix (Applied Biological Materials Inc, Richmond, Canada), 1.5 μL of synthesized cDNA product, 0.3 μL of each primer and 2.9 μL of ddH_2_O. The qRT-PCR reaction protocols were as follows: an enzyme activation step at 95 °C for 10 min with 1 cycle, denaturation at 95 °C for 15 s, and anneal/ extension at 60 °C for 60 s, for a total of 35 cycles. Technical samples and biological samples were used for all qRT-PCRs. Three biological replicates and technical repeats were used for each gene. The relative gene expression level was analyzed according to the 2^−ΔΔCt^ method [77].

## 5. Conclusions

This study was conducted to identify the *Hsp90* gene family in perennial ryegrass; hence, a genome-wide identification and expression analysis of the *LpHsp90* gene family was performed. Additionally, the gene structure, conserved motif, evolutionary relationships and expression patterns were studied. Eight *Hsp90* proteins were identified within the perennial ryegrass whole genome and were named according to their locations on the chromosomes. The sub-cellular localization of *LpHsp90* proteins indicated that they are mostly cytoplasmic. Two pairs of *LpHsp90* paralogous genes were identified (*LpHsp90-7* and *LpHsp90-8*, *LpHsp90-3* and *LpHsp90-5*) along with three orthologous gene pairs (*Bd4g06370* and *LpHsp90-4*, *Bd1g30130* and *LpHsp90-6*, *Bd4g32941* and *LpHsp90-2*). Expression pattens indicated that *LpHsp90-7*, *LpHs90-5*, *LpHs90-3* and *LpHsp90-1* were highly expressed under various stresses. *LpHsp90* proteins were generally highly expressed under heat stress and weakly under Cr stress. The functions of *LpHsp90* proteins remain unknown, and further studies are needed to determine their precise functions. This study provides a basis for future comprehensive studies on the functional analysis of *LpHsp90* proteins. Additionally, treatments such as MeJA and gibberellic acid would be of great interest in the experimental design and should be considered in future studies because they are important cellular regulators.

## Figures and Tables

**Table 1:** The biophysical characteristics and subcellular localization of *Lphsp90* proteins.

Gene	Molecular Weight	Theoretical pI	Number of Amino Acids	Instability Index	Predicted Sub-Cellular Location
*LpHsp90-1*	80,409.24	4.96	700	41.43	Cytoplasmic
*LpHsp90-2*	89,044.8	5.19	787	43.15	Cytoplasmic
*LpHsp90-3*	80,947.92	4.95	710	40.22	Cytoplasmic
*LpHsp90-4*	96,712.15	5.57	862	43.84	Nuclear
*LpHsp90-5*	61,214.61	5.08	528	44.14	Cytoplasmic
*LpHsp90-6*	92,834.85	4.89	809	37.71	Endoplasmic reticulum
*LpHsp90-7*	88,305.63	4.9	779	47.45	Cytoplasmic
*LpHsp90-8*	88,305.63	4.9	779	47.4	Cytoplasmic

**Table 2:** Primer information for *LpHsp90* genes.

Gene	Forward-Primer (5′-3′)	Reverse-Primer (5′-3′)
*Lphsp90-1*	ATCGTCTCTGACCGTGTTGT	AAGCATCACCAGGTCCTTGA
*Lphsp90-2*	GCACACTTCACAACAGAGGG	CTCGCCATCAAAGTCATCCG
*Lphsp90-3*	CATCATGGACAACTGCGAGG	GGCGTAGTCCTCCTTGTTCT
*Lphsp90-4*	AGGAGGTGTTTCTTCGGGAG	TGCAATGGTCCCAAGAGAGT
*Lphsp90-5*	TCGGAGTTCATCAGCTACCC	GCTCACCTCCTTCACCTTCT
*Lphsp90-6*	GCAAGGACTCGAAGCTCAAG	TTGATCTCCAGGACACGCTT
*Lphsp90-7*	GCCAATTGATGAGGTTGCCA	TCGCAGACCAACCAAACTTG
*Lphsp90-8*	GCGGAGGAGAAGTTCGAGTA	CATCCCAATGCCAGTGTCAG

## Data Availability

Not applicable.

## Supplementary Materials

The following are available online at https://www.mdpi.com/article/10.3390/plants10112509/s1, Table S1: Analysis of cis-acting element of *LpHsp90* genes in perennial ryegrass. Figure S1: Unrooted phylogenetic tree of 8(eight) *LpHsp90* proteins with annotated functions. The green color represented *Hsp90* proteins in *Arabidopsis thaliana*, red for *Oryza sativa,* violet for *Brachypodium distachyon* and blue for *Lolium perenne*. Figure S2: Details of motif logo and consensus. Figure S3: Amino acid sequence alignment of 8(eight) *LpHsp90* and the location of the C-terminal EEVD motif predicted. Figure S4: Heatmap showing the expression pattern of tested *LpHsp90* genes of perennial ryegrass under (a) heat (b) NaCl (c) Cd (d) ABA (e) PEG treatment respectively. The color scale indicates expression values normalized by TB tools formula.

## References

[1] Xu Y., Wang J., Bonos S.A., Meyer W.A., Huang B. (2018). Candidate Genes and Molecular Markers Correlated to Physiological Traits for Heat Tolerance in Fine Fescue Cultivars. Int. J. Mol. Sci..

[2] Huang B., Dacosta M., Jiang Y. (2014). Research advances in mechanisms of turfgrass tolerance to abiotic stresses: From physiology to molecular biology. Crit. Rev. Plant Sci..

[3] Huang L., Yan H., Jiang X., Yin G., Zhang X., Qi X., Zhang Y., Yan Y., Ma X., Peng Y. (2014). Identification of candidate reference genes in perennial ryegrass for quantitative RT-PCR under various abiotic stress conditions. PLoS ONE.

[4] Holmes W., Robson M.J., Parsons A.J., Williams T.E., Gill M., Beever D.E., Osbourn D.F., Murdoch J.C., Nix J.S., Green B.H. (2010). Grass: Its production and utilization. N. Z. J. Agric. Res..

[5] Wang W., Vinocur B., Shoseyov O., Altman A. (2004). Role of plant heat-shock proteins and molecular chaperones in the abiotic stress response. Trends Plant Sci..

[6] Nakashima K., Yamaguchi-Shinozaki K. (2006). Regulons involved in osmotic stress-responsive and cold stress-responsive gene expression in plants. Physiol. Plant..

[7] Chaves M.M., Maroco J.P., Pereira J.S. (2003). Understanding plant responses to drought—From genes to the whole plant. Funct. Plant Biol..

[8] Bailey-Serres J., Voesenek L. (2008). Flooding stress: Acclimations and genetic diversity. Annu. Rev. Plant Biol..

[9] Norris I.B. (2010). Relationships between growth and measured weather factors among contrasting varieties of *Lolium*, *Dactylis* and *Festuca* species. Grass Forage Sci..

[10] Ollerenshaw J.H. (1985). Influence of waterlogging on the emergence and growth of *Lolium perenne* L. shoots from seed coated with calcium peroxide. Plant Soil.

[11] Pearson A., Cogan N.O.I., Baillie R.C., Hand M.L., Bandaranayake C.K., Erb S., Wang J., Kearney G.A., Gendall A.R., Smithet K.F. (2011). Identification of QTLs for morphological traits influencing waterlogging tolerance in perennial ryegrass (*Lolium perenne* L.). Theor. Appl. Genet..

[12] Xiong Y., Fei S.Z., Arora R., Brummer E.C., Warnke S.E. (2007). Identification of quantitative trait loci controlling winter hardiness in an annual x perennial ryegrass interspecific hybrid population. Mol. Breed..

[13] Queitsch C., Sangster T.A., Lindquist S. (2002). *Hsp90* as a capacitor of phenotypic variation. Nature.

[14] Robert J. (2003). Evolution of heat shock protein and immunity. Dev. Comp. Immunol..

[15] Hsu A.L., Murphy C.T., Kenyon C. (2003). Regulation of aging and age-related disease by DAF-16 and heat-shock factor. Science.

[16] Krishna P. (2003). Plant responses to heat stress. Plant Responses to Abiotic Stress.

[17] Czar M.J., Galigniana M.D., Silverstein A.M., Pratt W.B. (1997). Geldanamycin, a Heat Shock Protein 90-Binding Benzoquinone Ansamycin, Inhibits Steroid-Dependent Translocation of the Glucocorticoid Receptor from the Cytoplasm to the Nucleus. Biochemistry.

[18] Pratt W.B., Krishna P., Olsen L.J. (2001). *Hsp90*-binding immunophilins in plants: The protein movers. Trends Plant Sci..

[19] Lindquist S. (1986). The Heat-Shock Response. Annu. Rev. Biochem..

[20] Iqbal N., Farooq S., Arshad R., Hameed A. (2010). Differential accumulation of high and low molecular weight heat shock proteins in basmati rice (*Oryza sativa* L.) cultivars. Genet. Resour. Crop. Evol..

[21] Lindquist S., Craig E.A. (1988). The heat-shock proteins. Annu. Rev. Genet..

[22] Tapia H., Morano K. (2010). *Hsp90* nuclear accumulation in quiescence is linked to chaperone function and spore development in yeast. Mol. Biol. Cell.

[23] Hashemi-petroudi S.H., Nematzadeh G., Mohammadi S., Kuhlmann M. (2019). Analysis of expression pattern of genome and analysis of *HSP90* gene family in *Aeluropus littoralis* under salinity stress. J. Crop. Breed..

[24] Chaudhary R., Baranwal V.K., Kumar R., Sircar D., Chauhan H. (2019). Genome-wide identification and expression analysis of *Hsp70*, *Hsp90*, and *Hsp100* heat shock protein genes in barley under stress conditions and reproductive development. Funct. Integr. Genom..

[25] Chen J., Gao T., Wan S., Zhang Y., Yang J., Yu Y., Wang W. (2018). Genome-Wide identification, classification and expression analysis of the *HSP* gene superfamily in tea plant (*Camellia sinensis*). Int. J. Mol. Sci..

[26] Zhang K., He S., Sui Y., Gao Q., Jia S., Lu X., Jia L. (2021). Genome-Wide Characterization of *HSP90* Gene Family in Cucumber and Their Potential Roles in Response to Abiotic and Biotic Stresses. Front. Genet..

[27] Zhang Z., Quick M.K., Kanelakis K.C., Gijzen M., Krishna P. (2003). Characterization of a plant homolog of hop, a cochaperone of *hsp90*. Plant Physiol..

[28] Yamano T., Mizukami S., Murata S., Chiba T., Tanaka K., Udono H. (2008). *Hsp90*-mediated assembly of the 26 S proteasome is involved in major histocompatibility complex class I antigen processing. J. Biol. Chem..

[29] Feng J., Fan P., Jiang P., Lv S., Chen X., Li Y. (2014). Chloroplast targeted *Hsp90* plays essential roles in plastid development and embryogenesis in *Arabidopsis* possibly linking with VIPP1. Physiol. Plant..

[30] Oh S.E., Yeung C., Babaei-Rad R., Zhao R. (2014). Cosuppression of the chloroplast localized molecular chaperone *HSP90.5* impairs plant development and chloroplast biogenesis in *Arabidopsis*. BMC Res. Notes.

[31] Reddy R.K., Chaudhary S., Patil P., Krishna P. (1998). The 90 kDa heat shock protein (*hsp90*) is expressed throughout *Brassica napus* seed development and germination. Plant Sci..

[32] Sable A., Rai K.M., Choudhary A., Yadav V.K., Agarwal S.K., Sawant S.V. (2018). Inhibition of heat shock proteins *HSP90* and *HSP70* induce oxidative stress, suppressing cotton fiber development. Sci. Rep..

[33] Hoffmann T., Hovemann B. (1988). Heat-shock proteins, *Hsp84* and *Hsp86*, of mice and men: Two related genes encode formerly identified tumour-specific transplantation antigens. Gene.

[34] Rebbe N.F., Ware J., Bertina R.M., Modrich P., Stafford D.W. (1987). Nucleotide sequence of a cDNA for a member of the human 90-kDa heat-shock protein family. Gene.

[35] Krone P.H., Sass J.B. (1994). *Hsp 90α* and *Hsp 90β* genes are present in the zebrafish and are differentially regulated in developing embryos. Biochem. Biophys. Res. Commun..

[36] Chen B., Zhong D., Monteiro A. (2006). Comparative genomics and evolution of the *HSP90* family of genes across all kingdoms of organisms. BMC Genom..

[37] Song H., Fan P., Li Y. (2009). Overexpression of organellar and cytosolic *AtHSP90* in *Arabidopsis thaliana* impairs plant tolerance to oxidative stress. Plant Mol. Biol. Rep..

[38] Hu W., Hu G., Han B. (2009). Genome-wide survey and expression profiling of heat shock proteins and heat shock factors revealed overlapped and stress specific response under abiotic stresses in rice. Plant Sci..

[39] Krishna P., Gloor G. (2001). The *Hsp90* family of proteins in *Arabidopsis thaliana*. Cell Stress Chaperones.

[40] Yang X., Zhu W., Zhang H., Liu N., Tian S. (2016). Heat shock factors in tomatoes: Genome-wide identification, phylogenetic analysis and expression profiling under development and heat stress. PeerJ.

[41] Yamada K., Fukao Y., Hayashi M., Fukazawa M., Nishimura M. (2007). Cytosolic *HSP90* regulates the heat shock response that is responsible for heat acclimation in *Arabidopsis thaliana*. J. Biol. Chem..

[42] McLellan C.A., Turbyville T.J., Wijeratne E.M.K., Kerschen A., Vierling E., Queitsch C., Whitesell L., Gunatilaka A.A.L. (2007). A rhizosphere fungus enhances Arabidopsis thermotolerance through production of an *HSP90* inhibitor. Plant Physiol..

[43] Takahashi A., Casais C., Ichimura K., Shirasu K. (2003). *HSP90* interacts with RAR1 and SGT1 and is essential for RPS2-mediated disease resistance in Arabidopsis. Proc. Natl. Acad. Sci. USA.

[44] Liu Y., Burch-Smith T., Schiff M., Feng S., Dinesh-Kumar S.P. (2004). Molecular chaperone *Hsp90* associates with resistance protein N and its signaling proteins SGT1 and Rar1 to modulate an innate immune response in plants. J. Biol. Chem..

[45] Tominaga H., Coury D.A., Amano H., Miki W., Kakinuma M. (2012). cDNA cloning and expression analysis of two heat shock protein genes, *Hsp90* and *Hsp60*, from a sterile *Ulva pertusa* (Ulvales, Chlorophyta). Fish. Sci..

[46] Sołtys-Kalina D., Szajko K., Sierocka I., Śliwka J., Strzelczyk-Żyta D., Wasilewicz-Flis I., Jakuczun H., Szweykowska-Kulinska Z., Marczewski W. (2015). Novel candidate genes AuxRP and *Hsp90* influence the chip color of potato tubers. Mol. Breed..

[47] Manchado M., Salas-Leiton E., Infante C., Ponce M., Asensio E., Crespo A., Zuasti E., Cañavate J.P. (2008). Molecular characterization, gene expression and transcriptional regulation of cytosolic *Hsp90*s in the flatfish Senegalese sole (*Solea senegalensis* Kaup). Gene.

[48] Galea-Lauri J., Latchman D.S., Katz D.R. (1996). The role of the 90-kDa heat shock protein in cell cycle control and differentiation of the monoblastoid cell line U937. Exp. Cell Res..

[49] Zhang H., Li L., Ye T., Chen R., Gao X., Xu Z. (2016). Molecular characterization, expression pattern and function analysis of the *OsHSP90* family in rice. Biotechnol. Biotechnol. Equip..

[50] Bai J., Pennill L.A., Ning J., Lee S.W., Ramalingam J., Webb C.A., Zhao B., Sun Q., Nelson J.C., Leach J.E. (2002). Diversity in nucleotide binding site-leucine-rich repeat genes in cereals. Genome Res..

[51] Han Y., Chen Y., Yin S., Zhang M., Wang W. (2015). Over-expression of TaEXPB23, a wheat expansin gene, improves oxidative stress tolerance in transgenic tobacco plants. J. Plant Physiol..

[52] Chory J., Wu D. (2001). Weaving the Complex Web of Signal Transduction. Plant Physiol..

[53] Mach J. (2009). Alternative splicing produces a JAZ protein that is not broken down in response to jasmonic acid. Plant Cell.

[54] Al-Whaibi M.H. (2011). Plant heat-shock proteins: A mini review. J. King Saud Univ. Sci..

[55] Genest O., Wickner S., Doyle S.M. (2019). *Hsp90* and *Hsp70* chaperones: Collaborators in protein remodeling. J. Biol. Chem..

[56] Milioni D., Hatzopoulos P. (1997). Genomic organization of *Hsp90* gene family in Arabidopsis. Plant Mol. Biol..

[57] Ritossa F. (1962). A new puffing pattern induced by temperature shock and DNP in drosophila. Experientia.

[58] Cha J., Ahn G., Kim J.Y., Kang S.B., Kim M.R., Su’udi M., Kim W.Y., Son D. (2013). Structural and functional differences of cytosolic 90-kDa heat-shock proteins (*Hsp90s*) in *Arabidopsis thaliana*. Plant Physiol. Biochem..

[59] Jackson S.E., Queitsch C., Toft D. (2004). *Hsp90*: From structure to phenotype. Nat. Struct. Mol. Biol..

[60] Shinozaki F., Minami M., Chiba T., Suzuki M., Yoshimatsu K., Ichikawa Y., Terasawa K., Emori Y., Matsumoto K., Kurosaki T. (2006). Depletion of *hsp90beta* induces multiple defects in B cell receptor signaling. J. Biol. Chem..

[61] Rehn A., Moroni E., Zierer B.K., Tippel F., Morra G., John C., Richter K., Colombo G., Buchner J. (2016). Allosteric Regulation Points Control the Conformational Dynamics of the Molecular Chaperone *Hsp90*. J. Mol. Biol..

[62] Zhang J., Li J., Liu B., Zhang L., Chen J., Lu M. (2013). Genome-wide analysis of the Populus *Hsp90* gene family reveals differential expression patterns, localization, and heat stress responses. BMC Genom..

[63] Song Z., Pan F., Yang C., Jia H., Jiang H., He F., Li N., Lu X., Zhang H. (2019). Genome-wide identification and expression analysis of *HSP90* gene family in *Nicotiana tabacum*. BMC Genet..

[64] Zai W.S., Miao L.X., Xiong Z.L., Zhang H.L., Ma Y.R., Li Y.L., Chen Y.B., Ye S.G. (2015). Comprehensive identification and expression analysis of *Hsp90s* gene family in *Solanum lycopersicum*. Genet. Mol. Res..

[65] Kawahara Y., de la Bastide M., Hamilton J.P., Kanamori H., McCombie W.R., Ouyang S., Schwartz D.C., Tanaka T., Wu J., Zhou S. (2013). Improvement of the *Oryza sativa* Nipponbare reference genome using next generation sequence and optical map data. Rice.

[66] Liu Y., Wan H., Yang Y., Wei Y., Li Z., Ye Q., Wang R., Ruan M., Yao Z., Zhou G. (2014). Genome-wide identification and analysis of heat shock protein 90 in tomato. Yi Chuan.

[67] Jain M., Tyagi A.K., Khurana J.P. (2006). Genome-wide analysis, evolutionary expansion, and expression of early auxin-responsive SAUR gene family in rice (*Oryza sativa*). Genomics.

[68] Skolnick J., Fetrow J.S. (2000). From genes to protein structure and function: Novel applications of computational approaches in the genomic era. Trends Biotechnol..

[69] Singh R.K., Lee J.K., Selvaraj C., Singh R., Li J., Kim S.Y., Kalia V.C. (2018). Protein Engineering Approaches in the Post-Genomic Era. Curr. Protein Pept. Sci..

[70] Jin Z., Chandrasekaran U., Liu A. (2014). Genome-wide analysis of the Dof transcription factors in castor bean (*Ricinus communis* L.). Genes Genom..

[71] Byrne S.L., Nagy I., Pfeifer M., Armstead I., Asp T. (2015). A synteny-based draft genome sequence of the forage grass *Lolium perenne*. Plant J..

[72] Larkin M.A. (2007). Clustal W and Clustal X version 2.0. Bioinformatics.

[73] Guidon S., Gascuel O. (2003). A simple, fast and accurate algorithm to estimate large phylogenies by maximum likelihood. Syst. Biol..

[74] Tamura K., Stecher G., Peterson D., Filipski A., Kumar S. (2013). MEGA6: Molecular Evolutionary Genetics Analysis version 6.0. Mol. Biol. Evol..

[75] Hu B., Jin J., Guo A.Y., Zhang H., Luo J., Gao G. (2014). GSDS 2.0: An upgraded gene feature visualization server. Bioinformatics.

[76] Bailey T.L., Boden M., Buske F.A., Frith M., Grant C.E., Clementi L., Ren J., Li W.W., Noble W.S. (2009). MEME SUITE: Tools for motif discovery and searching. Nucleic Acids Res..

[77] Schmittgen T.D., Livak K.J. (2008). Analyzing real-time PCR data by the comparative C(T) method. Nat. Protoc..

