# Supplementary figures and images for: Genome-Wide Identification of Hsp90 Gene Family in Perennial Ryegrass and Expression Analysis under Various Abiotic Stresses
Source: Plants (Basel). 2021 Nov 19;10(11):2509. doi: 10.3390/plants10112509 (PMC8622807; doi:10.3390/plants10112509)

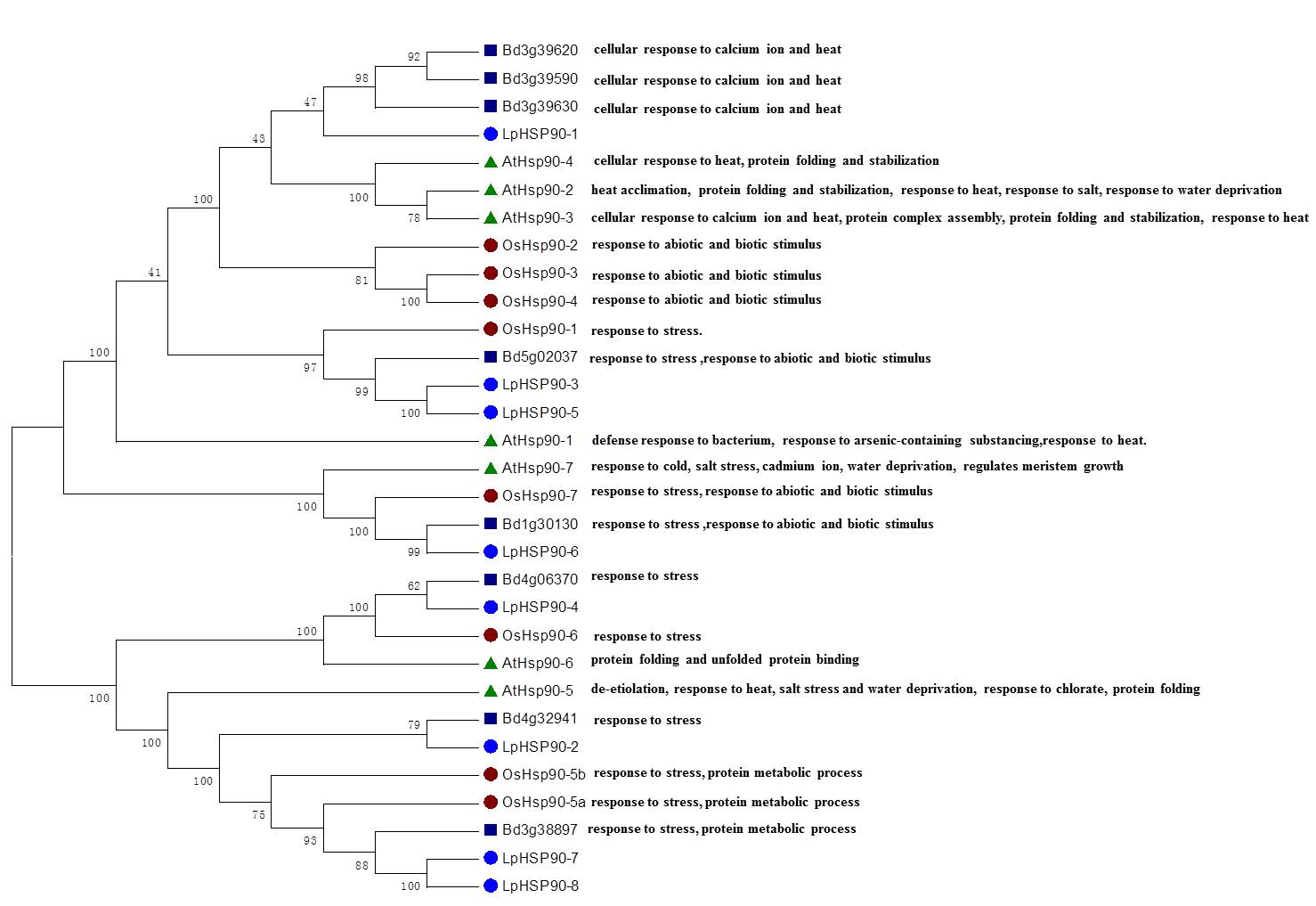

Supplement: Supplementary file 1 [file plants-10-02509-s001.zip › Supplementory files/Figure S1 Function.jpg]

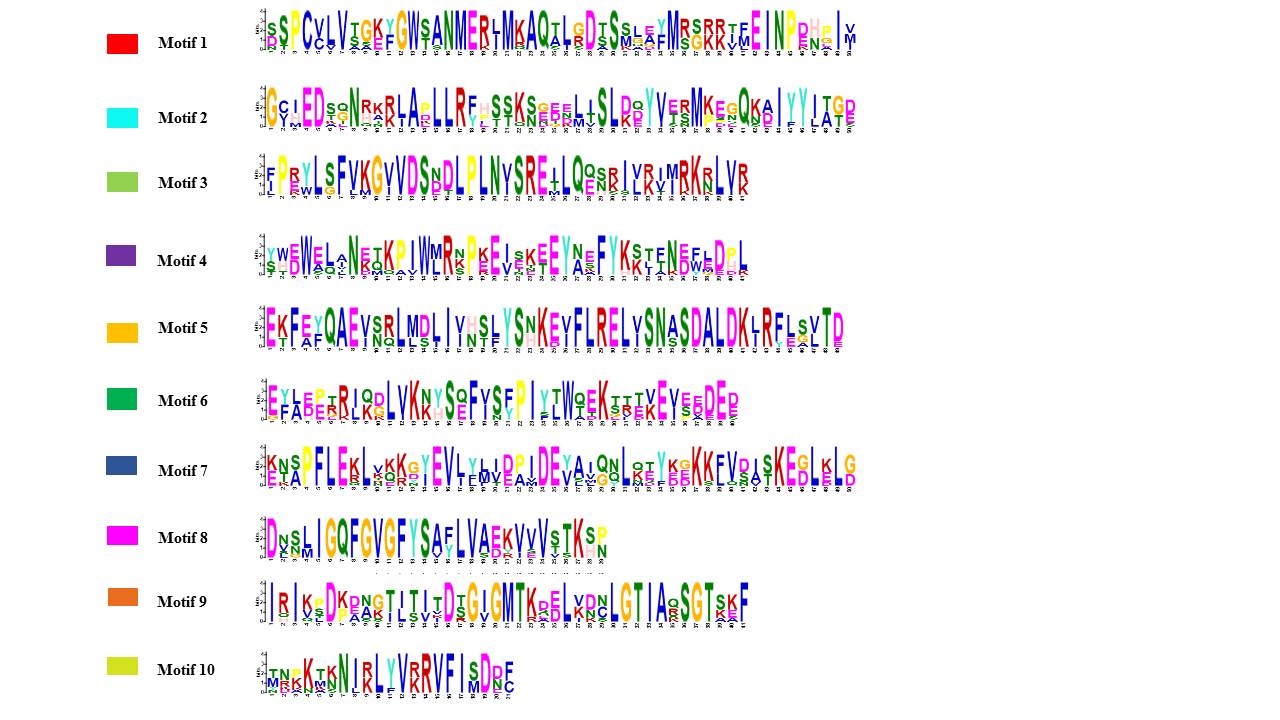

Supplement: Supplementary file 1 [file plants-10-02509-s001.zip › Supplementory files/Figure S2 Sequence logo of LpHsp90 protein seqences.jpg]

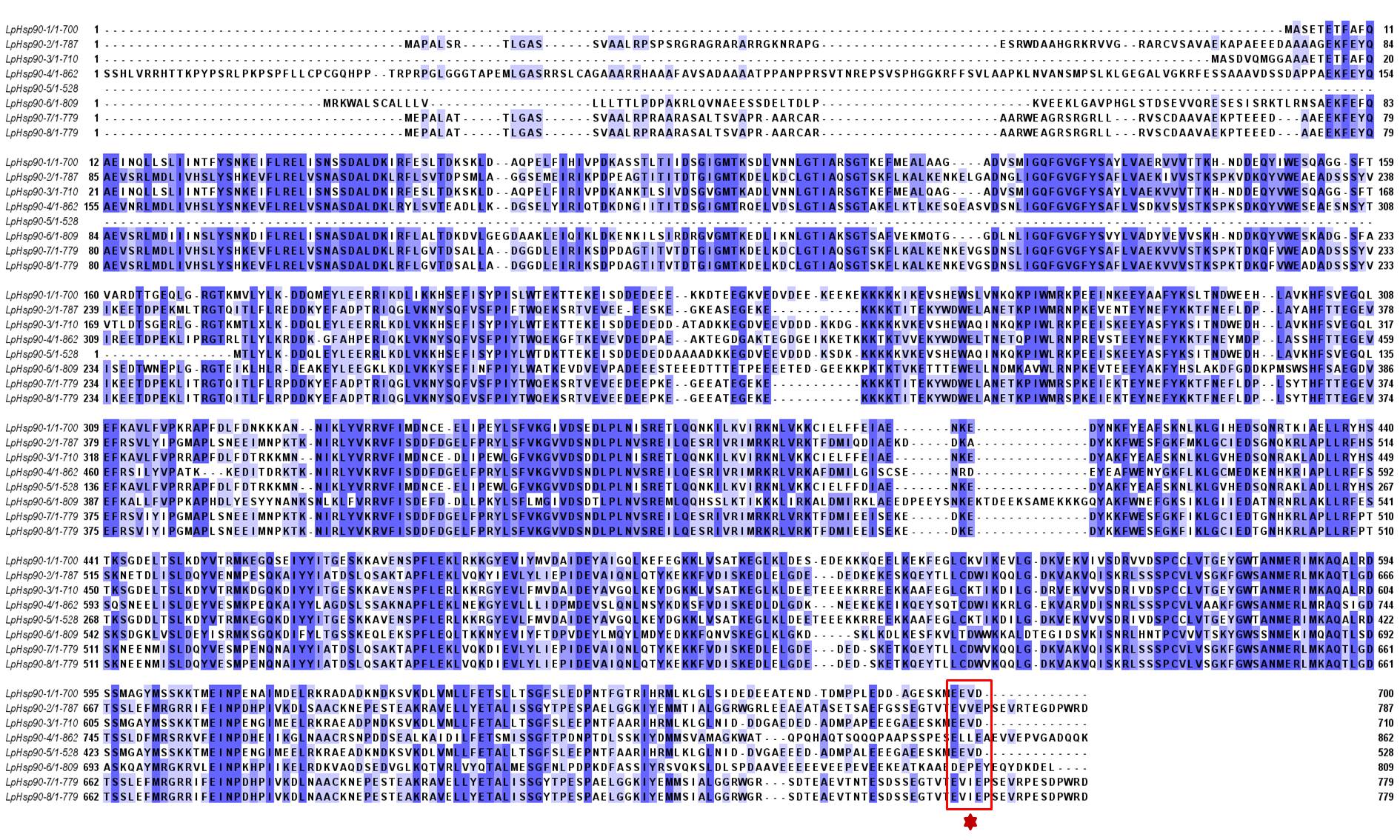

Supplement: Supplementary file 1 [file plants-10-02509-s001.zip › Supplementory files/Figure S3.jpg]

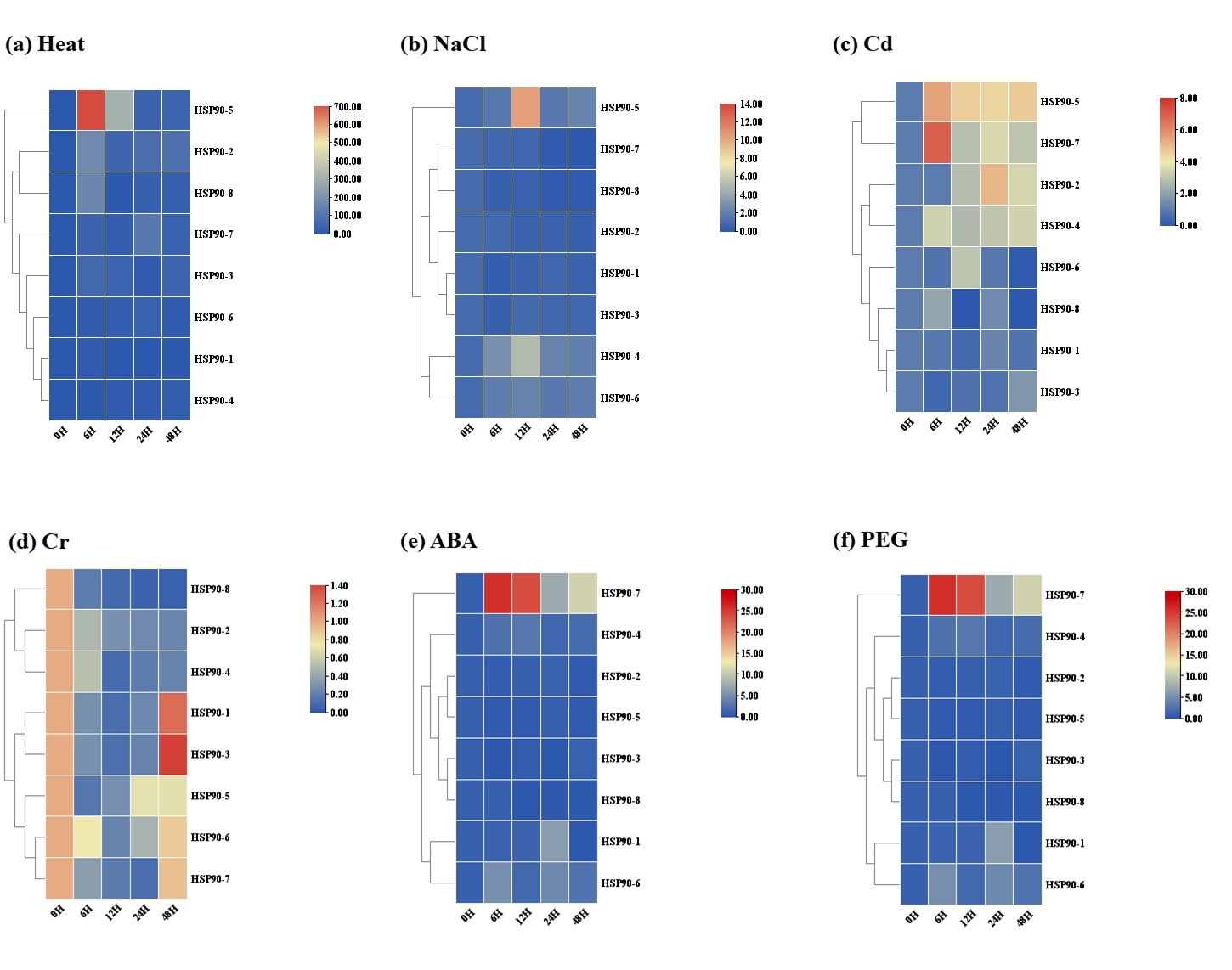

Supplement: Supplementary file 1 [file plants-10-02509-s001.zip › Supplementory files/Figure S4.tif]
